# Metabolomics of cerebrospinal fluid reveals prognostic biomarkers in pediatric status epilepticus

**DOI:** 10.1111/cns.14312

**Published:** 2023-06-28

**Authors:** Tianqi Wang, Chunpei Li, Yu Ma, Hao Zhou, Xiaonan Du, Yingfeng Li, Shasha Long, Yifeng Ding, Guoping Lu, Weiming Chen, Yuanfeng Zhou, Lifei Yu, Ji Wang, Yi Wang

**Affiliations:** ^1^ Department of Neurology, National Children's Medical Center Children's Hospital of Fudan University Shanghai China; ^2^ Department of Developmental Behavioral Pediatrics, Guizhou Provincial People's Hospital Medical College of Guizhou University Guiyang China; ^3^ Pediatric Intensive Care Unit, National Children's Medical Center Children's Hospital of Fudan University Shanghai China

**Keywords:** cerebrospinal fluid, children, metabolomics, prognostic biomarker, status epilepticus

## Abstract

**Aims:**

Status epilepticus (SE) is the most common neurological emergency in pediatric patients. This study aimed to screen for prognostic biomarkers of SE in the cerebrospinal fluid (CSF) using metabolomics.

**Methods:**

Ultra‐performance liquid chromatography quadrupole time‐of‐flight tandem mass spectrometry (UPLC‐QTOF‐MS) was conducted to identify prognostic biomarkers in CSF metabolomics by comparing the poor outcome group (*N* = 13) with the good outcome group (*N* = 15) of children with SE. Differentially expressed metabolites were identified using Mann–Whitney *U* test corrected by Benjamini‐Hochberg and partial least squares discriminant analysis (PLS‐DA).

**Results:**

The PLS‐DA model identified and validated significant metabolic differences between the poor and good outcome groups of children with SE (PLS‐DA with *R* ^2^Y = 0.992 and *Q* ^2^ = 0.798). A total of 49 prognosis‐related metabolites were identified. Of these metabolites, 20 including glutamyl‐glutamine, 3‐iodothyronamine, and L‐fucose had an area under the curve (AUC) ≥ 80% in prognostic prediction of SE. The logistic regression model combining glutamyl‐glutamine and 3‐iodothyronamine produced an AUC value of 0.976, with a sensitivity of 0.863 and specificity of 0.956. Pathway analysis revealed that dysregulation of the citrate cycle (TCA) and arginine biosynthesis may contribute to poor SE prognosis.

**Conclusions:**

This study highlighted the prognosis‐related metabolomic disturbances in the CSF of children with SE and identified potential prognostic biomarkers. A prognostic prediction model combining glutamyl‐glutamine and 3‐iodothyronamine with high predictive value was established.

## Graphical abstract

Status epilepticus (SE) could induce metabolic changes in brain and could be reflected in cerebrospinal fluid (CSF). Metabolomic analysis using ultra‐performance liquid chromatography quadrupole time‐of‐flight tandem mass spectrometry (UPLC‐QTOF‐MS) was applied to screen potential prognostic biomarkers in CSF of children with SE, by comparing the poor outcome group with the good outcome group.

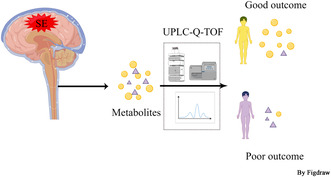

## 1 INTRODUCTION

Status epilepticus (SE) is the most common neurological emergency in pediatric patients. SE is defined as abnormally prolonged seizures that can have long‐term consequences, including neuronal injury, neuronal death, and alteration of networks.^1^ It is estimated that children suffer from 17 to 23 SE episodes per 100,000 per year, 0%–6% of which can progress to death.^2^, ^3^, ^4^ Despite increasing therapeutic options and evolving treatment protocols in recent years, 14%–24% of children still have neurological dysfunction.^5^, ^6^

In patients with SE, early prognostic assessment is critical for both treatment and early rehabilitation interventions. Prognostic factors including demographic and clinical characteristics, electroencephalography (EEG), and brain magnetic resonance imaging (MRI) have been combined for SE outcome prediction.^7^ A six point outcome prediction score named PEDSS (pre‐SE pediatric cerebral performance category score, background EEG abnormalities, drug refractoriness, semiology, and critical sickness) showed high predictive accuracy for mortality.^8^ However, the predictability of poor outcomes especially brain injury, in pediatric SE still needs to be improved. Biochemical markers, such as blood glucose, serum aspartate aminotransferase, C‐reactive protein, serum albumin, and procalcitonin have been reported to be associated with poor outcomes in children with SE.^9^, ^10^, ^11^

Although a correlation between biochemical markers has been found, the predictive value of those biomarkers is closely associated with the etiology of severe bacterial infections that are not specific to SE.^9^ New prognostic biomarkers with high specificity for SE that reflect neuronal injury needed to be identified. However, brain molecular disturbances in SE are difficult to manifest in the blood because most of them cannot penetrate the blood–brain barrier. Thus, cerebrospinal fluid (CSF) is the promising medium for identifying new potential biomarkers of central nervous system diseases. CSF is anatomically in close contact with the brain parenchyma and contains neural products whose changes may reflect different brain function states.^12^ Additionally, CSF biomarkers of Alzheimer's disease and traumatic brain injury perform well in predicting functional outcomes and serving as therapeutic targets.^13^, ^14^

Metabolomics is a technology that profiles the metabolites in biofluids (serum, urine, tears, saliva, and CSF), cells, and tissues to reveal subtle alterations in biological pathways under physiological and pathological conditions.^15^ It has proven useful in quantitatively and multiparametrically assessing organismal responses to development, environmental perturbation, exogenous exposure, and disease response,^15^ Because of its ability to identify multiple biochemicals, non‐targeted metabolic profiling analysis is routinely used as an initial tool for biomarker discovery.^16^ By applying nontargeted metabolomics, Boguszewicz et al.^17^ identified biomarkers of drug‐resistant epilepsy in children, including serum N‐acetyl‐glycoprotein, lactate, creatine, glycine, and lipids. Additionally, evolving statistical technologies and metabolome databases have enabled the use of non‐targeted metabolomics to discover new risk factors, diagnostic and prognostic markers, and drug targets.^18^ Ultra‐performance liquid chromatography quadrupole time‐of‐flight tandem mass spectrometry (UPLC‐QTOF‐MS) is a non‐targeted metabolomic technology with sensitive detection and relatively reliable quantitation abilities.^19^ Considering the brain metabolites disturbed in SE, if prognostic metabolites could be identified before an irreversibly poor outcome, it would be helpful to guide clinical decisions and prevent poor prognosis.^20^

In this study, we used UPLC‐QTOF‐MS to explore prognosis‐related metabolites by comparing the poor and good outcome groups of children with SE. This study attempted to elucidate the prognosis‐related metabolic mechanisms and identify potential prognostic prediction biomarkers for SE.

## 2 METHODS

### 2.1 Study participants and cerebrospinal fluid samples

This study prospectively enrolled patients who were admitted to the Children's Hospital of Fudan University and diagnosed with SE between January 1, 2017, and December 31, 2021. The definition of SE was tonic–clonic seizures ≥5 min, focal seizures with impaired consciousness ≥10 min, or absence seizures ≥15 min according to the International League Against Epilepsy (ILAE) guidelines.^1^

The inclusion criteria for the SE group were: (1) children diagnosed with SE aged 29 days to 18 years, (2) lumbar puncture for CSF detection was necessary according to professional clinicians, and (3) informed consent was obtained from the parents or guardians of the children to recover the remaining CSF samples after biochemical testing. The exclusion criteria included: (1) the interval between lumbar puncture and SE onset exceeding 72 h, (2) obvious hemolysis in CSF samples, (3) remaining CSF sample volume < 50 μL, and (4) the interval between retention and recovery exceeded 4 h. The inclusion criteria for the non‐SE control group were: (1) children aged 29 days to 18 years, (2) new‐onset hematologic disease without neurological symptoms, (3) lumbar puncture for CSF detection was necessary to rule out CNS involvement according to the clinicians, and (4) informed consent was obtained from the parents or guardians of the children to recover the remaining CSF samples after the biochemical testing. The exclusion criteria included:(1) CFS test suggested neurological involvement, (2) the interval between lumbar puncture and SE onset exceeding 72 h, (3) obvious hemolysis in CSF samples, (4) remaining CSF sample volume < 50 μL, and (5) the interval between retention and recovery exceeding 4 h.

### 2.2 Outcome classification

Outcomes were assessed at discharge using the Pediatric Cerebral Performance Category (PCPC) and Glasgow Outcome Scale‐Extended Pediatric version (GOS‐E Peds).^21^, ^22^ Consistent with a previous study, poor outcome was defined as ≥1 increase from pre‐admission to discharge in the PCPC score and/or GOS‐E Peds, whereas good outcome was defined as no increase in the score.^23^ Clinical features, including age, sex, diagnosis, SE duration, seizure type, etiology, and CSF test results, were collected. This study was conducted in accordance with the principles of the Declaration of Helsinki. The study was approved by the Ethics Committee of the Children's Hospital of Fudan University.

### 2.3 Chemicals and reagents

HPLC‐grade methanol and ammonium formate were purchased from Sigma‐Aldrich. Formic acid and acetonitrile were purchased from Thermo Fisher Scientific. Methyl tert‐butyl ether was purchased from Aladdin Co, Ltd.

### 2.4 Sample preparation

The CSF samples were separately packed into EP tubes (50 μL per tube), labeled, and stored at −80°C. At the beginning of the metabolomics analysis, the CSF samples thawed slowly at 4°C. Then, 20 μL thawed CSF sample was mixed with 100 μL pre‐cooled methanol for protein precipitation. Samples were vortex‐mixed and centrifuged at 14,000 *g*, 4°C for 10 min, then, 90 μL of supernatant was extracted and added to 250 μL pre‐cooled methyl tert‐butyl ether solution and vortex‐mixed. Next, 45 μL of ultrapure water was added. The solution was mixed and centrifuged at 14,000 *g*, 4°C for 5 min. The bottom layer was transferred to a sample vial and analyzed. The quality control (QC) samples were generated by pooling and mixing all the samples.

### 2.5 Metabolic profiling

#### 2.5.1 Mass spectral condition

A Water Xevo G2‐XS QTOF (Waters Corp.) was used in full MS scan mode for data acquisition between 50 and 1000 m/z, with a scan time of approximately 0.1 s. The following parameters were then set: capillary voltages of 2500 V and 2000 V for positive and negative ion electrospray modes, source temperature of 120°C, desolvation temperature of 500°C, and desolvation gas flow of 800 L/h.

#### 2.5.2 Chromatographic condition

The ACQUITY I‐Class UPLC (Water Crop.) fitted with a T3 (Waters ACQUITY UPLC HSS T3, 2.1 × 100 mm, 1.8 μm) and Amide Column (Waters ACQUITY UPLC BEH Amide, 2.1 × 100 mm, 1.7 μm) was used. For the T3 Column, mobile phases A and B were water/formic acid (99.9:0.1) and acetonitrile/formic acid (99.9:0.1), respectively. The column temperature, flow rate, and injection volume were 40°C, 0.5 mL/min, and 2 μL, respectively. For the Amide Column, mobile phase A comprised of acetonitrile: water, 3% formic acid, and 10 mM aqueous ammonium formate (5:95). Mobile phase B comprised of water: acetonitrile, 3% formic acid, and 10 mM aqueous ammonium formate (5:95). The column temperature, flow rate, and injection volume were 45°C, 0.6 mL/min, and 2 μL, respectively.

### 2.6 Data processing and statistical analysis

Metabolomic features were preprocessed using Progenesis QI software (Waters, Nonlinear Dynamics) for peak detection, alignment, and area deconvolution. Then the data were log‐transformed and normalized to a list containing the mass charge ratio (m/z), retention time (RT), and peak identity. Metabolite identification was performed by matching accurate m/z, isotope, and MS/MS spectra to public databases including METLIN, Human Metabolomics Database (HMDB), and LipidBlast. The data were imported into SIMCA‐P software (Version 14.1) for the unsupervised method, principal component analysis (PCA), and partial least squares discriminant analysis (PLS‐DA) to identify differences in CSF metabolic profiles between the good and poor outcome SE, and control groups. Variable importance projection (VIP) values > 1 in the PLS‐DA model were selected because they represented a more significant influence on group differentiation. The Mann–Whitney *U* test was performed to analyze the differences between the two groups, and the false discovery rate (FDR) was calculated using Benjamini‐Hochberg correction. Metabolites with *p* values < 0.05, FDR < 0.2, and VIP values > 1 were considered statistically significant in this study.^24^, ^25^ A fold change (FC) analysis was also performed to calculate the FC value. Logistic regression analysis and receiver operating characteristic (ROC) curves were used to calculate the area under the curve (AUC) of the biomarkers and analyze the prediction model. Metabolic pathway analyses were performed using MetaboAnalyst (Version 5.0). The Wilcoxon rank‐sum test, Kruskal‐Wallis test, and Pearson Chi‐square test were used to compare continuous and categorical variables and determine the statistical significance of differences between groups. Differences were considered statistically significant at *p* value < 0.05.

## 3 RESULTS

### 3.1 Clinical characters of participants

A total of 28 children diagnosed with SE were enrolled in this study, including 13 with poor and 15 with good outcomes. There were no significant differences in age, sex, biochemical characteristics of CSF, or etiology between the two groups (*p* > 0.05). The prognosis varied according to SE severity (Table 1). Nine children enrolled in the non‐SE control group were comparable in terms of age and sex (*p* > 0.05).

**TABLE 1:** Demographics of children in the SE and the non‐SE groups.

Demographic	Non‐SE group (*N* = 9)	SE group			*p* Value (three groups)
		Good outcome (*N* = 15)	Poor outcome (*N* = 13)	*p* Value	
Age, year	4.4 (1.7, 13.8)	3.1 (0.2, 14.1)	6.7 (0.4, 13)	0.394	0.370
Sex					
Male	2 (22.2)	10 (66.7)	7 (53.8)	0.488	0.112
Female	7 (77.8)	5 (33.3)	6 (46.2)		
SE severity					
SE	/	9 (60.0)	2 (15.4)	0.040	
RSE	/	4 (26.7)	5 (38.5)		
SRSE	/	2 (13.3)	6 (46.1)		
CSF					
Glucose (mmol/L)	3.5 (3.0, 4.7)	3.4 (3.1, 5.0)	3.7 (2.9, 5.7)	0.379	0.392
Cl (mmol/L)	125 (122, 129)	127 (123, 132)	128 (119, 136)	0.595	0.613
Protein (mg/L)	149.5 (69, 272.3)	350 (124, 509.3)	335 (141.9, 297.5)	0.300	0.009
Etiology					
Acute symptomatic	/	9 (60.0)	10 (76.9)	0.548	
Unknown	/	5 (33.3)	2 (15.4)		
Others	/	1 (6.7)	1 (7.1)		

Abbreviations: CSF, cerebrospinal fluid; RSE, refractory status epilepticus; SE, status epilepticus; SRSE, super‐refractory status epilepticus.

After instrumental analysis, peak detection, alignment, and peak deconvolution, 3409 features were included for metabolite identification, and 439 known metabolites were identified. After removing repetitive and exogenous metabolites, 221 metabolites were analyzed. We assessed the quality of the metabolic data in the dataset. The PCA results revealed that the QC samples clustered together, indicating that the detection process was repeatable and robust (Figure S1).

### 3.2 Cerebrospinal fluid metabolic profiling of status epilepticus groups with different outcomes

**FIGURE 1:**
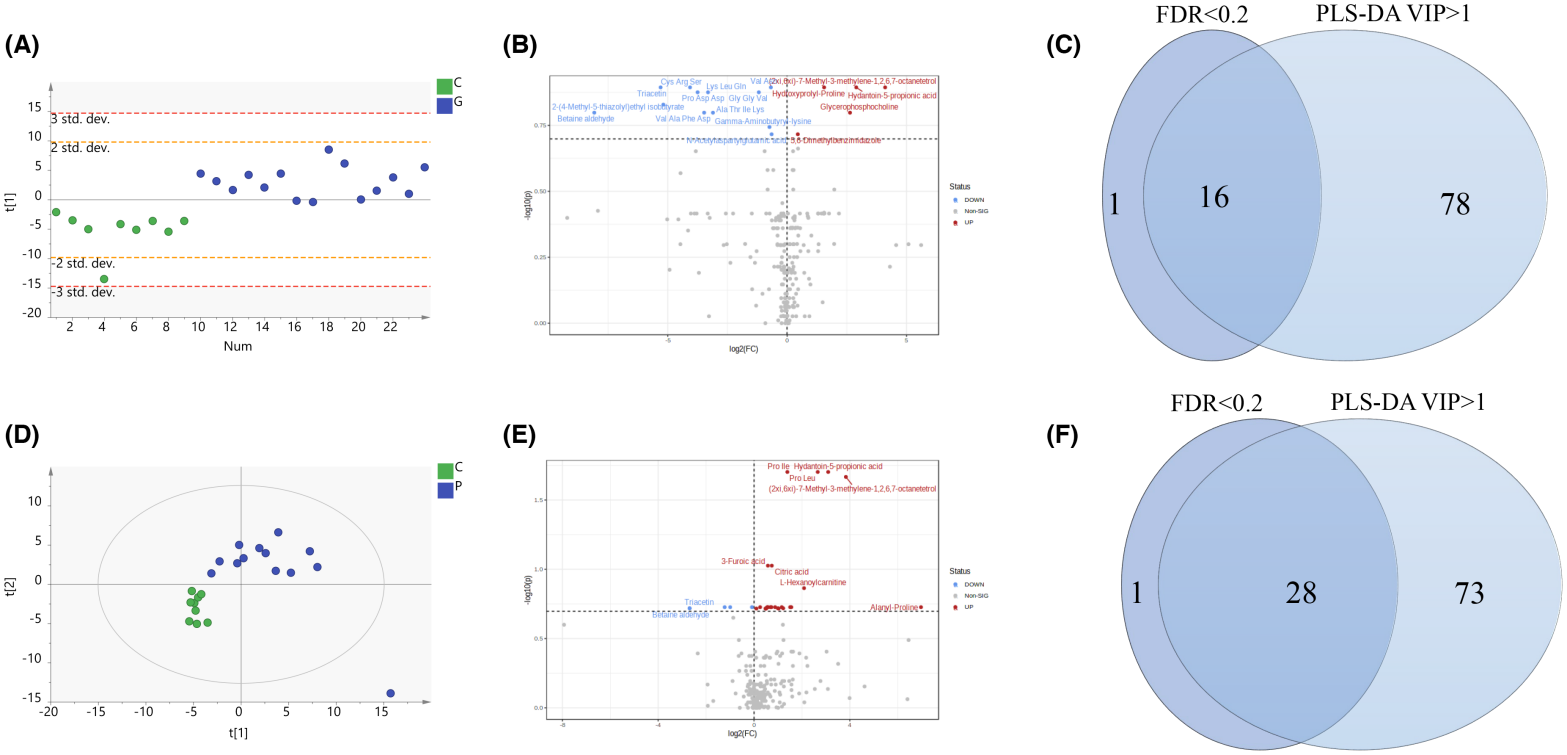
Altered metabolic profiles in the status epilepticus (SE) with good and poor outcome groups compared with the non‐SE group. (A) Partial least squares discriminant analysis (PLS‐DA) scores scatter plot of the SE with good outcome and non‐SE groups. *R* ^2^X = 0.127, *R* ^2^Y = 0.679, and *Q* ^2^ = 0.405. CV‐ANOVA test: *p* = 0.004, *F* = 7.149. (B) Volcano plot of the differential metabolites in cerebrospinal fluid (CSF) of the SE with good outcome group filtered by false discovery rate (FDR) analysis. (C) Venn diagram of the differential metabolites in the SE with good outcome group filtered by the PLS‐DA model and FDR analysis. (D) The PLS‐DA scores scatter plot of the SE with poor outcome and non‐SE groups. *R* ^2^X = 0.386, *R* ^2^Y = 0.926, and *Q* ^2^ = 0.632. CV‐ANOVA test: *p* = 0.016, *F* = 3.843. One sample in the SE with poor outcome group was an outlier. (E) Volcano plot of the differential metabolites in CSF of the SE with poor outcome group filtered by FDR analysis. (F) Venn diagram of the differential metabolites in the SE with good outcome group filtered by the PLS‐DA model and an FDR analysis.

To characterize the metabolomic features of the SE group with good outcomes, a comparative study involving patients with SE with good outcomes and a non‐SE control group was performed. To comprehensively identify differential metabolites, we constructed a PLS‐DA model (*R* ^2^Y = 0.679, *Q* ^2^ = 0.405) and compared it with the non‐SE group. The CSF metabolites of children with SE with good outcomes could be differentiated from those of the control group, and the CV‐ANOVA (*p* = 0.004, *F* = 7.149) test suggested that the model was not overfitted. Metabolites with VIP values > 1 (94 metabolites) according to the PLS‐DA model and FDR < 0.2 (17 metabolites) according to the Mann–Whitney *U* test with Benjamini‐Hochberg correction were selected. As presented in the Venn diagram, 16 metabolites significantly changed in the CSF of children with SE with good outcomes (Figure 1, Table S1). Of the altered metabolites, the levels of four metabolites including (2′,6′)‐7‐methyl‐3‐methylene‐1,2,6,7‐octanetetrol, hydantoin‐5‐propionic acid, hydroxyprolyl‐proline and glycerophosphocholine were significantly increased. Twelve metabolites, including triacetin, betaine aldehyde, and gamma‐aminobutyryl‐lysine, decreased. The different metabolites were enriched in alanine, aspartate and glutamate metabolism, and glycerophospholipid metabolism.

The metabolomic signature of the SE group with poor outcomes was also characterized by a comparative study involving SE patients with poor outcomes and a non‐SE control group. The CSF metabolites of children with SE with poor outcomes could be differentiated from those of the control group in the PLS‐DA model (*R* ^2^Y = 0.926, *Q* ^2^ = 0.632), and the CV‐ANOVA test (*p* = 0.016, *F* = 3.843) suggested that the model was not overfitted. Metabolites with VIP values > 1 (101 metabolites) according to the PLS‐DA model and FDR < 0.2 (29 metabolites) according to Mann–Whitney *U* test with Benjamini‐Hochberg correction were selected (Figure 1). As shown in Figure 1F, 28 significant different metabolites were identified. The levels of 23 metabolites, such as hydantoin‐5‐propionic acid, citrate, and carbamoyl phosphate were increased in the CSF of SE patients with poor outcomes compared to those in the non‐SE group. The levels of five metabolites diethylphosphate, L‐fucose, and betaine aldehyde, cytidine, and triacetin were significantly decreased (Table S2).

Based on the differential metabolites, we further screened the altered metabolic pathways in patients with SE with poor outcomes. Dysregulation of arginine biosynthesis; alanine, aspartate, and glutamate metabolism; and the citrate cycle (TCA) were found in children with SE with poor outcomes.

### 3.3 Identification of prognosis biomarkers of status epilepticus

**FIGURE 2:**
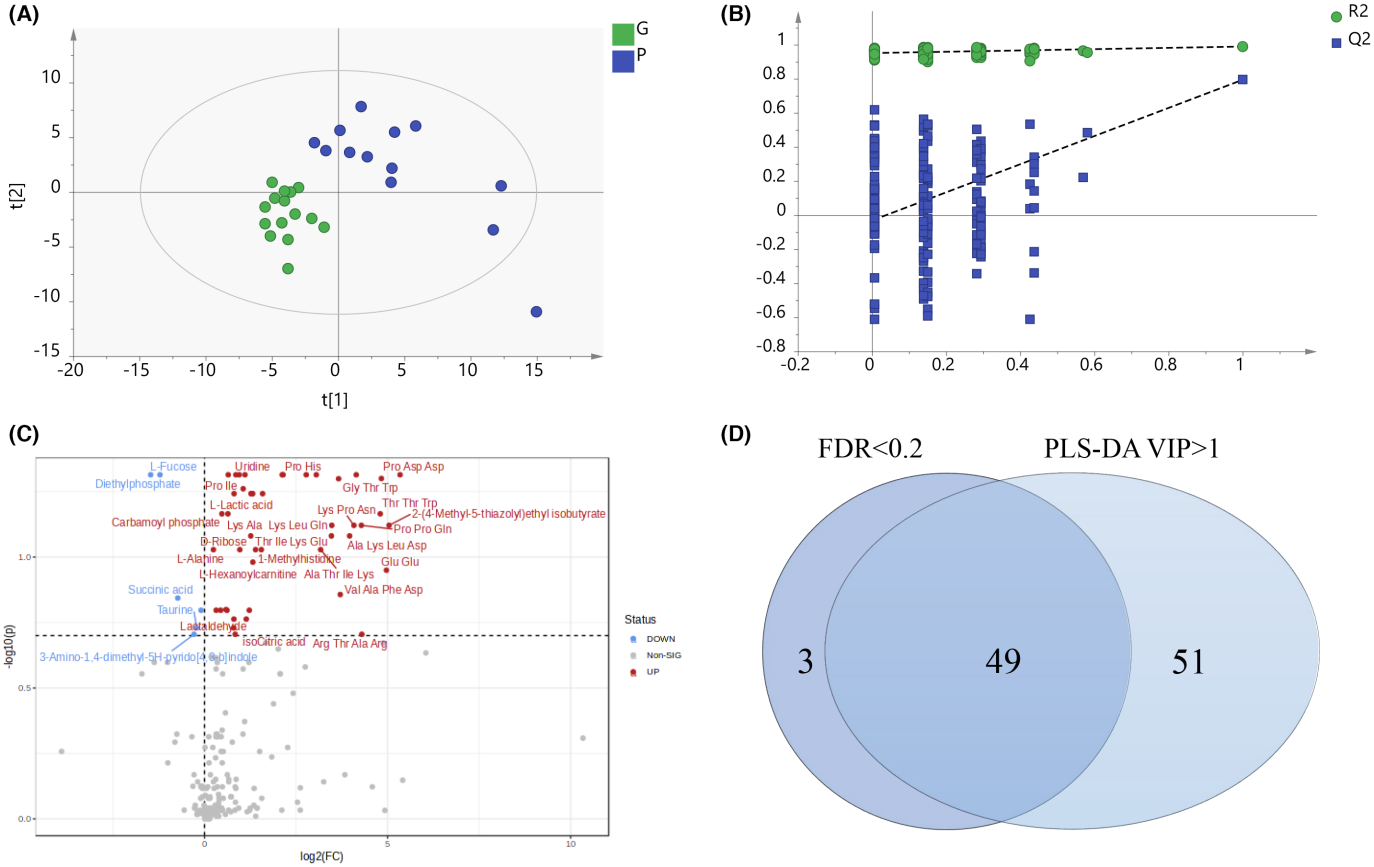
Comparison of the altered metabolic profiles in the status epilepticus (SE) with poor and good outcome groups. (A) Partial least squares discriminant analysis (PLS‐DA) scores scatter plot of the SE with poor and good outcome groups. *R* ^2^X = 0.531, *R* ^2^Y = 0.992, and *Q* ^2^ = 0.798. CV‐ANOVA test: *p* = 0.007, *F* = 3.820. One sample in the SE with poor outcome group was an outlier. (B) Permutation test (200 times) of the PLS‐DA model. *R* ^2^ = 0.954, *Q* ^2^ = −0.028. (C) Volcano plot of the differential metabolites in cerebrospinal fluid of SE with poor outcome group filtered by false discovery rate (FDR) analysis. (D) Venn diagram of the differential metabolites in the SE with poor outcome group filtered by the PLS‐DA model and FDR analysis.

To screen for prognostic biomarkers, we conducted a comparative study between poor and good outcome groups of children with SE. The PLS‐DA model clearly differentiated the two groups, with *R* ^2^Y = 0.992 and *Q* ^2^ = 0.798 (Figure 2A). The permutation test (*R* ^2^ = 0.954, *Q* ^2^ = −0.028) and CV‐ANOVA (*p* = 0.007, *F* = 3.820) suggested that the model was not overfitted (Figure 2B). Metabolites with *p* < 0.05, FDR <0.2 according to the Mann–Whitney *U* test (52 metabolites) with Benjamini‐Hochberg correction, and VIP > 1 (100 metabolites) according to the PLS‐DA model were selected (Figure 2C,D). A total of 49 metabolites were significantly altered in the CSF of children with poor outcomes compared with those with good outcomes (Table S3). Of these metabolites, 44, including glutamyl‐glutamine, lysyl‐glutamine, 3‐iodothyronamine, and uridine were significantly increased in the CSF of children with poor outcomes. Five metabolites decreased in children with poor outcome: diethylphosphate, L‐fucose, varanic acid, taurine, and succinate.

### 3.4 Receiver operating characteristic curve analysis

**FIGURE 3:**
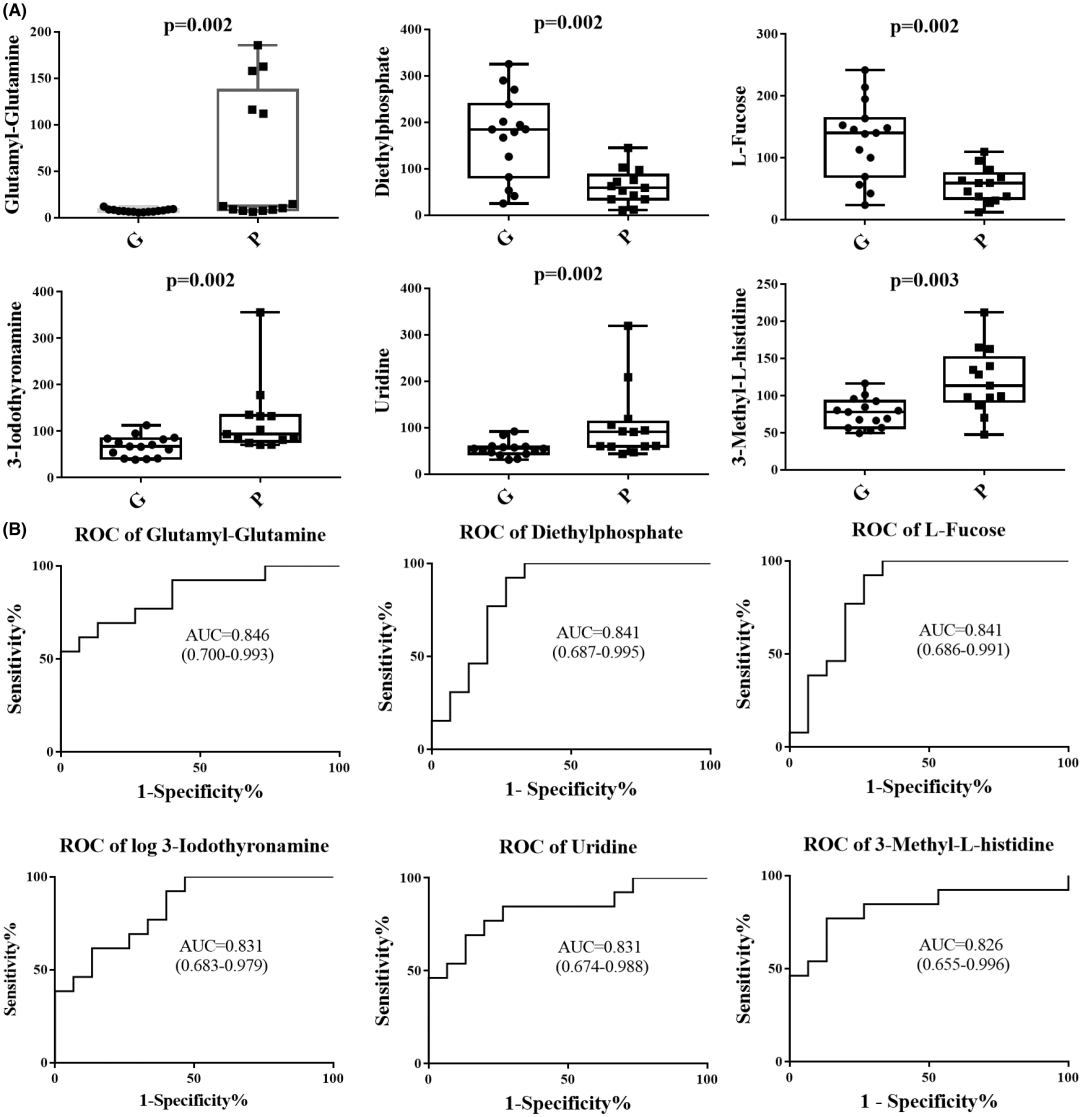
Potential prognostic biomarkers of status epilepticus (SE). (A) Box plots of the six major differential metabolites for the comparison between poor outcome and good outcome group of SE. Concentration of glutamyl‐glutamine showed a bimodal plot in the poor outcome group. (B) Receiver operating characteristic (ROC) curves illustrate the classification performance of the six prognostic biomarkers. The area under the curve (AUC), 95% CI of AUC, and specificities of each biomarker are indicated.

**FIGURE 4:**
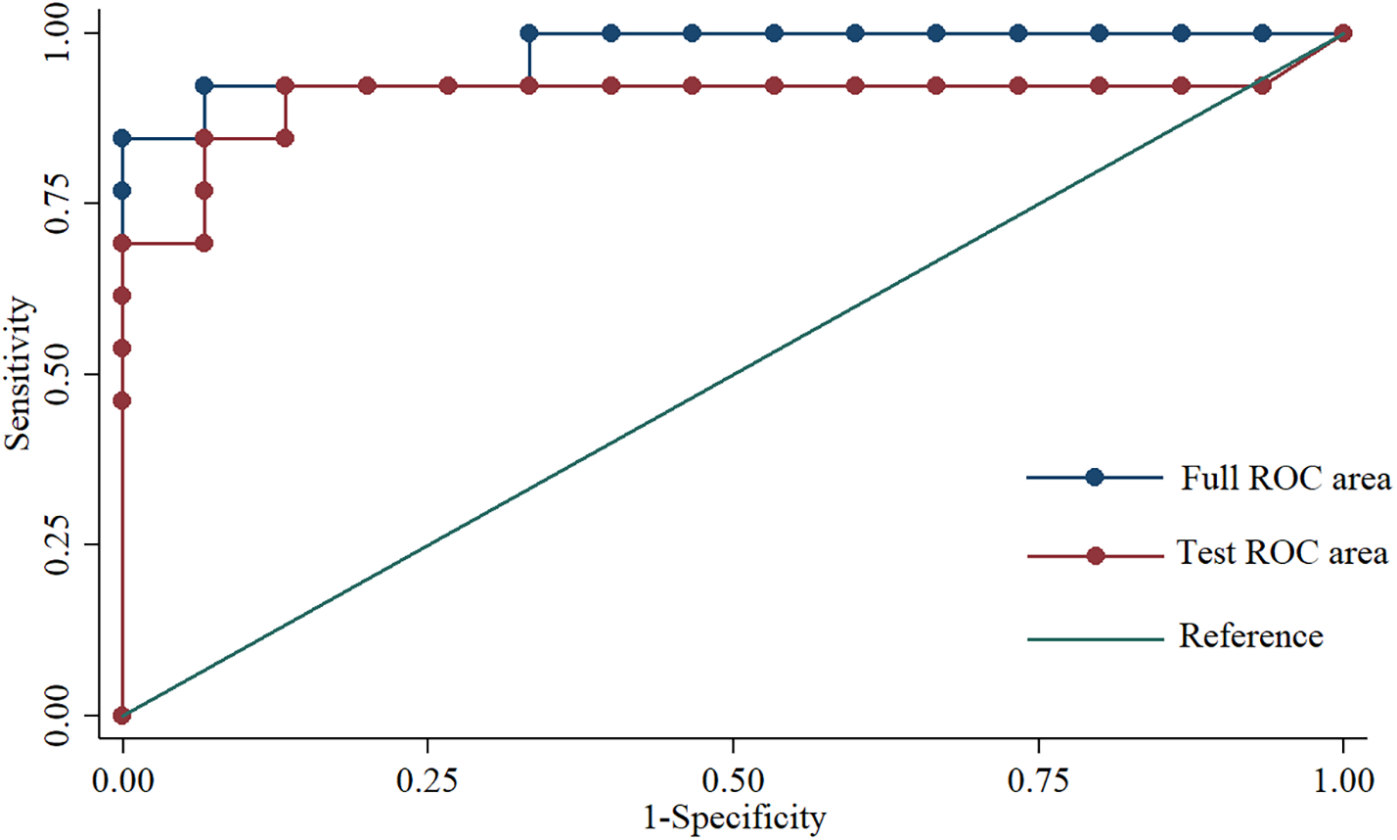
The receiver operating characteristic (ROC) curve of combined glutamyl‐glutamine and 3‐iodothyronamine discriminate poor from good outcomes in children with status epilepticus. The full ROC area was 0.97 (95% CI: 0.962–0.991), with the sensitivity and specificity of 0.863 and 0.956, respectively. The 10‐fold cross‐validation tested an ROC area of 0.910 (95% CI: 0.763–1.000), with a sensitivity of 0.923 and a specificity of 0.933.

To further evaluate the prognostic prediction performance of the identified metabolites, we performed univariate and multivariate ROC curve analyses to distinguish children with SE with poor outcomes from those with good outcomes. According to the classical univariate ROC curve classification models, 20 metabolites had an AUC ≥ 80% in prognostic prediction of SE. The levels of six major metabolites glutamyl‐glutamine, diethylphosphate, and L‐fucose, 3‐iodothyronamine, uridine, and 3‐methyl‐L‐histidine were plotted (Figure 3A). The ROC curves of the six metabolites were plotted with an AUC ranging from 0.826 to 0.846 (Figure 3B). Logistic regression multivariate ROC curve analysis combining glutamyl‐glutamine and 3‐iodothyronamine produced an AUC value of 0.976 (95% CI: 0.962–0.991), with a sensitivity of 0.863 and a specificity of 0.956. The 10‐fold cross‐validation tested the performance of the logistic regression model with an AUC value of 0.910 (95% CI: 0.763–1.000), with a sensitivity of 0.923, and a specificity of 0.933 (Figure 4).

### 3.5 Perturbed metabolic pathway

**FIGURE 5:**
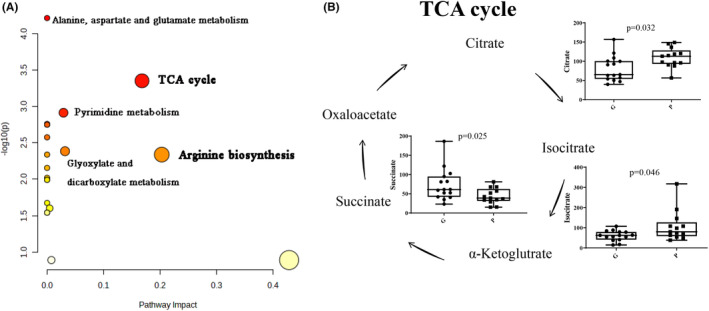
Integration of altered metabolic pathways for status epilepticus (SE) with poor outcome. (A) Ingenuity pathway analysis based on 49 significantly differential metabolites in the SE with poor outcome group compared with the good outcome group. (B) The citrate cycle metabolism.

Pathway analysis was performed to understand the functions of the prognostic metabolites we identified. The altered species associated with SE prognosis were mainly enriched in the TCA cycle; arginine biosynthesis; pyrimidine metabolism; glyoxylate and dicarboxylate metabolism; and alanine, aspartate, and glutamate metabolism (Figure 5).

## 4 DISCUSSION

This study is the first to present a comprehensive metabolic evaluation of the CSF of children with SE and to identify 49 prognosis‐related metabolites. Potential prognostic biomarkers, such as glutamyl‐glutamine, 3‐iodothyronamine, and L‐fucose showed good predictive performance. A prognostic prediction model consisting of glutamyl‐glutamine and 3‐iodothyronamine with satisfactory sensitivity and specificity was established. Metabolic disturbances in TCA cycle and the arginine biosynthesis may be involved in the pathogenesis of poor SE outcomes.

Metabolic disturbance was found in both the good and poor outcome groups of children with SE, indicating that the metabolic changes caused by SE could be reflected in the CSF. Metabolic disturbance appear more severe in children with poor outcomes, which may be related to increased oxidative stress, inflammation, or neuronal apoptosis.^26^ Altered metabolites and pathways could be involved in the pathogenesis and prognosis of SE, making it possible and reliable to search for biomarkers through metabolomics.

In this study, CSF metabolites were significantly altered in children with SE with poor outcomes compared with those with good outcomes, enabling the early prognostic prediction of SE. Among the prognostic biomarkers, most were increased in the CSF of children with poor outcomes compared with those with good outcomes. Glutamyl‐glutamine is a dipeptide composed of glutamate and glutamine and belongs to the family of N‐acyl‐alpha‐amino acids and derivatives. The increase in glutamyl‐glutamine levels may be related to glutamate and glutamine metabolism in SE, and the related physiological or cell‐signaling functions need to be studied. Additionally, the bimodal plot of glutamyl‐glutamine levels in children with poor outcomes may have been caused by different etiologies, durations of SE, and individual differences. Future studies with larger sample sizes should be conducted to explore the bimodal plot mechanism. Further, 3‐iodothyronamine, an endogenous thyroid hormone derivative that activates trace amine‐associated receptor 1,^27^ also increased in children with poor outcomes. The activation of apoptosis by inducing the expression of SIRT6 and inhibiting the mTOR pathway by 3‐iodothyronamine was found in a transgenic model of Alzheimer's disease.^28^ In children with SE, 3‐iodothyronamine may aggregate neuronal apoptosis, leading to a poor prognosis. Attenuation of the excessive expression of 3‐iodothyronamine or antagonizing its function may be potential treatments for improving the prognosis of SE.

Additionally, L‐fucose levels were significantly decreased in children with SE with poor outcomes. L‐fucose is an endogenous and exogenous monosaccharide found in mammals that appears to be involved in various biological functions. Previous studies reported the anti‐inflammation of L‐fucose in several systems such as the respiratory, endocrine, and digestive systems.^29^, ^30^ L‐fucose could inhibit macrophage cell M1 polarization, nitrogen permease regulator‐like 3 (NPRL3) inflammasome, and release of TNF‐α, IL‐1β, and IL‐6 pro‐inflammation cytokines, thus exerting anti‐inflammatory effect.^31^ Decreased L‐fucose levels could attenuate the anti‐inflammatory effect in children with SE, thus contributing to poor outcomes.

In this study, TCA cycle‐related metabolites, including citrate, isocitrate, and succinate, were significantly altered in children with poor outcomes, suggesting that dysregulation of aerobic energy metabolism is associated with the prognosis of SE. Dysregulation of the TCA cycle has been associated with several diseases related to oxidative stress, such as atrial fibrillation, heart failure,^32^ diabetic kidney disease,^33^ and neurodegenerative disease.^34^ In an in vitro model of amyotrophic lateral sclerosis, oxidative stress disrupted the concentrations of succinate and malate in motor neurons, indicating an impaired TCA cycle in disease physiology.^35^ TCA cycle intermediates, including citrate and α‐ketoglutarate, show significantly higher concentrations in Alzheimer's disease.^36^ Hippocampal mitochondrial dysregulation, oxidative stress, and cell death were observed in pilocarpine‐induced SE models.^37^, ^38^ TCA cycle dysregulation may be associated with neuronal excitability, gliosis, inflammation, and apoptosis in children with SE with poor outcomes. Additionally, metabolic treatments such as triheptanoin and a ketogenic diet have shown beneficial protection against SE and/or associated damage.^39^, ^40^ Supplementation with TCA cycle intermediates and preservation of TCA cycle metabolism in SE might be potential treatments to improve the prognosis.

The arginine biosynthesis pathway is also altered in the CSF of children with poor outcomes. As a precursor of nitric oxide (NO), arginine is significantly altered in neurological diseases, including schizophrenia, bipolar disorder, and migraine.^41^, ^42^ The potential anti‐inflammatory function of arginine has been reported previously.^43^ Excessive arginine biosynthesis may be an endogenous response to severe inflammation in children with SE with poor outcomes.

This study identified endogenous and exogenous metabolites in the CSF of children with SE. To identify prognostic biomarkers that could reflect the pathophysiology of SE, we excluded the exogenous metabolites and analyzed only the endogenous metabolites. This study had several limitations. One potential limitation in analyzing prognostic biomarkers was the small sample size. Although we identified promising prognostic biomarkers, further external validation is required to evaluate the sensitivity and specificity of these biomarkers. More specifically, targeted analyses of CSF metabolites in the future will help better characterize metabolic profiles and predict the prognosis of SE. Further, despite the restriction of the interval between lumbar puncture and SE onset to 72 h, metabolic profiling could still be affected by the puncture time for the real‐time characteristics of metabolomics. Additionally, the effects of pharmacological and non‐pharmacological treatments on metabolism were not analyzed in this study. The control group was also not healthy because of the traumatic nature of lumbar puncture. CSF metabolomics is still at risk of being affected by blood changes even after excluding CNS involvement.

## 5 CONCLUSION

In summary, we highlighted the prognosis‐related metabolic disturbances in the CSF of children with SE. Disturbances in the TCA cycle may contribute to the pathogenesis of a poor prognosis in SE. The prognosis of SE in children can be effectively predicted using a model that combines with glutamyl‐glutamine and 3‐iodothyronamine. Further validation studies in larger cohorts are necessary to determine the role of glutamyl‐glutamine and 3‐iodothyronamine in SE prognosis. These evaluations could improve our understanding of SE pathogenesis and facilitate biomarker screening for outcome predictions.

## AUTHOR CONTRIBUTIONS

Tianqi Wang designed the study, collected samples, conducted the experiments, analyzed the data, drafted and revised the article. Chunpei Li and Yu Ma collected samples, analyzed the data, draft and revised the article. Hao Zhou, Xiaonan Du, Yingfeng Li, Shasha Long and Yifeng Ding helped with samples collection and the data analysis. Weiming Chen, Guoping Lu, Yuanfeng Zhou and Lifei Yu helped with the design the study and revised the draft. Ji Wang and Yi Wang as the corresponding author of this manuscript designed the study and finally approved the version. All authors read and approved the final manuscript.

## FUNDING INFORMATION

This work was supported by the Project supported by Shanghai Municipal Science and Technology Major Project (Grant No. 2017SHZDZX01), Omics‐based precision medicine of epilepsy being entrusted by Key Research Project of the Ministry of Science and Technology of China (Grant No. 2016YFC0904400), and Shanghai Municipal Science and Technology Major Project (No. 2018SHZDZX01) and ZJLab.

## CONFLICT OF INTEREST STATEMENT

The authors declare no competing financial conflict of interest.

## Supporting information


Figure S1.

Tables S1–S3.

## Data Availability

All processed data used in this study can be obtained from the corresponding author on reasonable request.
