# Supplementary figures and images for: Metabolomics of cerebrospinal fluid reveals prognostic biomarkers in pediatric status epilepticus
Source: CNS Neurosci Ther. 2023 Jun 28;29(12):3925–34. doi: 10.1111/cns.14312 (PMC10651953; doi:10.1111/cns.14312)

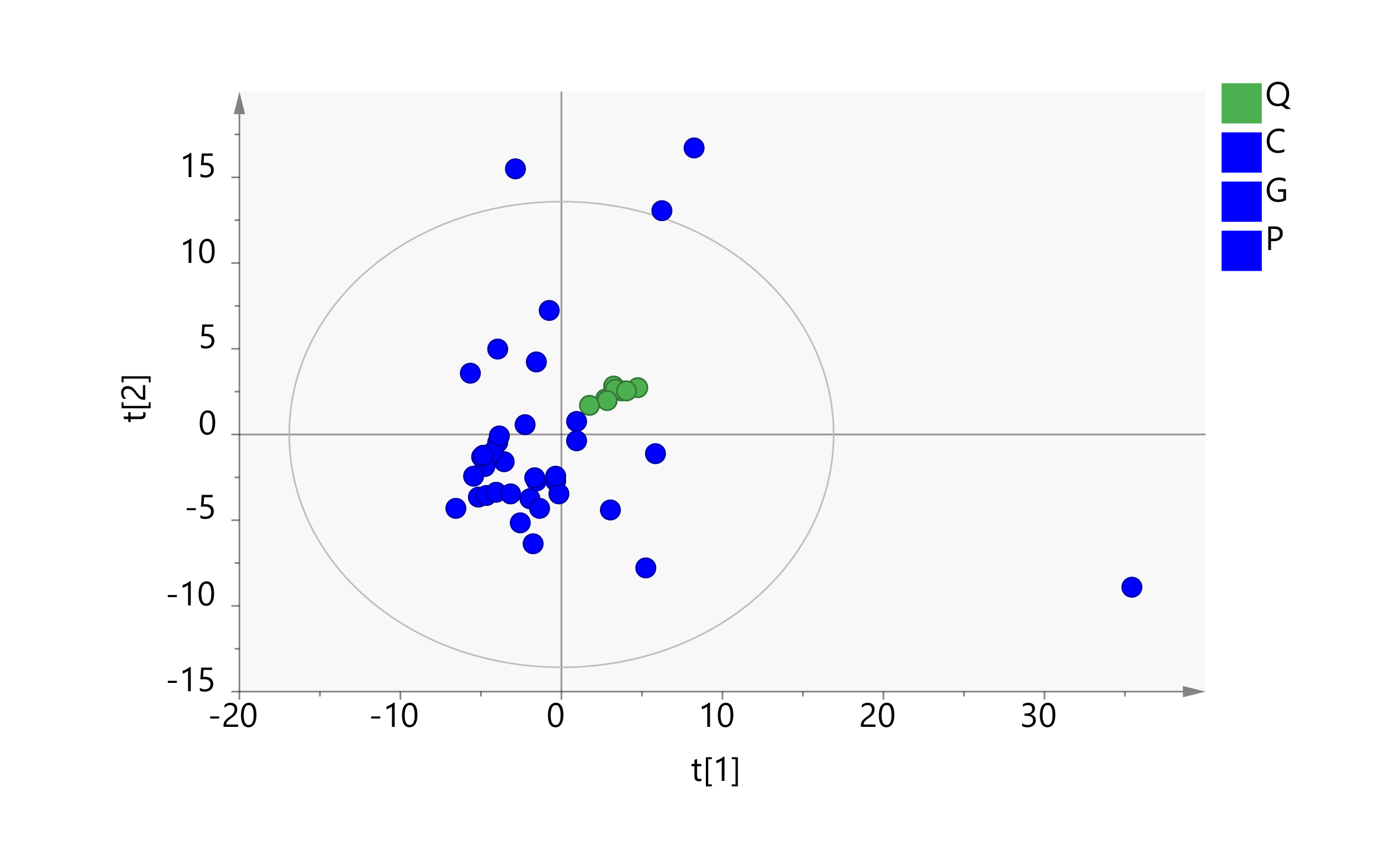

Supplement: Supplementary file 1 — Figure S1. [file CNS-29-3925-s001.tif]
