# Supplementary material for: Metabolomics of cerebrospinal fluid reveals prognostic biomarkers in pediatric status epilepticus
Source: CNS Neurosci Ther. 2023 Jun 28;29(12):3925–34. doi: 10.1111/cns.14312 (PMC10651953; doi:10.1111/cns.14312)
Supplement: Supplementary file 2 — Tables S1–S3. [file CNS-29-3925-s002.docx]

**Table S1 Differential metabolites between SE with good outcome and non-SE control group**

| Compounds | m/z | Retention time (min) | p value | FDR | VIP | FC |
| --- | --- | --- | --- | --- | --- | --- |
| Hydroxyprolyl-Proline | 229.1185 | 0.5877 | 0.001 | 0.128 | 1.931 | 2.928 |
| Cys Arg Ser | 365.1576 | 0.7245 | 0.002 | 0.128 | 1.350 | 0.060 |
| (2xi,6xi)-7-Methyl-3-methylene-1,2,6,7-octanetetrol | 205.1425 | 0.5182 | 0.002 | 0.128 | 1.205 | 17.219 |
| Val Asn | 232.1293 | 5.7972 | 0.003 | 0.128 | 2.170 | 0.624 |
| Hydantoin-5-propionic acid | 217.0476 | 0.5211 | 0.003 | 0.128 | 1.588 | 7.441 |
| Triacetin | 241.0697 | 2.9054 | 0.003 | 0.128 | 2.486 | 0.025 |
| Gly Gly Val | 232.1294 | 0.5649 | 0.004 | 0.133 | 2.116 | 0.439 |
| Pro Asp Asp | 346.1230 | 0.5097 | 0.005 | 0.133 | 1.457 | 0.074 |
| Lys Leu Gln | 388.2547 | 0.7245 | 0.005 | 0.133 | 1.322 | 0.101 |
| 2-(4-Methyl-5-thiazolyl)ethyl isobutyrate | 214.0897 | 0.5215 | 0.007 | 0.148 | 1.696 | 0.027 |
| Val Ala Phe Asp | 451.2152 | 4.2158 | 0.009 | 0.159 | 1.211 | 0.090 |
| Betaine aldehyde | 102.0917 | 1.5079 | 0.009 | 0.159 | 2.276 | 0.004 |
| Glycerophosphocholine | 258.1106 | 5.7124 | 0.010 | 0.159 | 1.523 | 6.205 |
| Ala Thr Ile Lys | 432.2808 | 0.7245 | 0.010 | 0.159 | 1.276 | 0.116 |
| Gamma-Aminobutyryl-lysine | 232.1659 | 6.1488 | 0.012 | 0.180 | 1.783 | 0.598 |
| N-Acetylaspartylglutamic acid | 303.0836 | 4.7720 | 0.015 | 0.192 | 1.575 | 0.637 |

Abbreviations: SE, status epilepticus; FDR, false discovery rate; FC, fold change; VIP, variable importance projection.

**Table S2 Differential metabolites between SE with poor outcome and non-SE control group**

| Compounds | m/z | Retention time (min) | p value | FDR | VIP | FC |
| --- | --- | --- | --- | --- | --- | --- |
| Pro Leu | 229.1551 | 4.3478 | 0.000 | 0.020 | 1.658 | 6.322 |
| Pro Ile | 229.1551 | 3.9085 | 0.000 | 0.020 | 1.878 | 2.624 |
| Hydantoin-5-propionic acid | 217.0476 | 0.5211 | 0.000 | 0.020 | 1.540 | 8.551 |
| (2xi,6xi)-7-Methyl-3-methylene-1,2,6,7-octanetetrol | 205.1425 | 0.5182 | 0.000 | 0.022 | 1.304 | 14.282 |
| 3-Furoic acid | 111.0083 | 1.2131 | 0.003 | 0.094 | 2.086 | 1.494 |
| Citrate | 191.0190 | 1.2131 | 0.003 | 0.094 | 2.064 | 1.674 |
| L-Hexanoylcarnitine | 260.1858 | 1.6871 | 0.004 | 0.137 | 1.251 | 4.255 |
| Carbamoyl phosphate | 185.9807 | 0.5021 | 0.007 | 0.188 | 1.500 | 1.501 |
| Lys Pro Leu | 357.2499 | 5.9535 | 0.009 | 0.188 | 1.563 | 1.840 |
| Glutarylcarnitine | 276.1446 | 4.3287 | 0.009 | 0.188 | 1.333 | 2.942 |
| Diethylphosphate | 199.0374 | 3.1399 | 0.011 | 0.188 | 1.664 | 0.428 |
| Alanyl-Proline | 209.0897 | 2.7796 | 0.012 | 0.188 | 1.309 | 125.310 |
| 3-Methyl-L-histidine | 170.0929 | 5.9459 | 0.014 | 0.188 | 1.432 | 1.655 |
| 4-Hydroxy-2-butenoic acid gamma-lactone | 85.0286 | 3.1366 | 0.014 | 0.188 | 1.247 | 2.230 |
| (R)-3-Hydroxybutyric acid | 103.0398 | 1.2430 | 0.014 | 0.188 | 1.168 | 2.845 |
| Palmitic amide | 256.2634 | 0.4323 | 0.017 | 0.188 | 1.857 | 1.194 |
| L-Fucose | 209.0667 | 3.1439 | 0.017 | 0.188 | 1.575 | 0.502 |
| (R)-(+)-2-Pyrrolidone-5-carboxylic acid | 130.0504 | 5.5523 | 0.017 | 0.188 | 1.425 | 1.464 |
| Cytidine | 266.0749 | 0.8736 | 0.017 | 0.188 | 1.205 | 0.942 |
| ADMA | 203.1506 | 5.8544 | 0.017 | 0.188 | 1.093 | 1.608 |
| Triacetin | 241.0697 | 2.9054 | 0.021 | 0.191 | 1.603 | 0.155 |
| Betaine aldehyde | 102.0917 | 1.5079 | 0.021 | 0.191 | 1.401 | 0.155 |
| D-Ribose | 149.0447 | 0.5250 | 0.021 | 0.191 | 1.305 | 1.998 |
| Acetylcarnitine | 204.1236 | 3.1327 | 0.021 | 0.191 | 1.237 | 2.327 |
| Acetylcarnosine | 313.1131 | 5.5707 | 0.025 | 0.192 | 1.467 | 1.401 |
| L-Glutamine | 145.0615 | 5.5707 | 0.025 | 0.192 | 1.438 | 1.395 |
| D-Glutamine | 147.0770 | 5.5523 | 0.025 | 0.192 | 1.423 | 1.424 |
| Tiglylcarnitine | 244.1546 | 2.0892 | 0.025 | 0.192 | 1.382 | 2.036 |

Abbreviations: SE, status epilepticus; FDR, false discovery rate; FC, fold change; VIP, variable importance projection.

**Table S3 Differential metabolites between SE with poor outcome and good outcome group**

| Compounds | m/z | Retention time (min) | p value | FDR | VIP | FC | AUC |
| --- | --- | --- | --- | --- | --- | --- | --- |
| Lysyl-Glutamine | 297.1551 | 2.7659 | 0.001 | 0.048 | 1.370 | 4.352 | 0.846 |
| Glutamyl-Glutamine | 275.1104 | 3.3104 | 0.002 | 0.048 | 1.383 | 8.299 | 0.846 |
| Diethylphosphate | 199.0374 | 3.1399 | 0.002 | 0.048 | 1.749 | 0.360 | 0.841 |
| L-Fucose | 209.0667 | 3.1439 | 0.002 | 0.048 | 1.696 | 0.430 | 0.841 |
| Pro His | 275.1106 | 2.9183 | 0.002 | 0.048 | 1.382 | 6.859 | 0.836 |
| Gly Thr Trp | 363.1629 | 3.8908 | 0.003 | 0.050 | 1.373 | 28.467 | 0.833 |
| 3-Iodothyronamine | 354.0023 | 0.9110 | 0.002 | 0.048 | 1.311 | 1.811 | 0.831 |
| Uridine | 267.0591 | 0.9346 | 0.002 | 0.048 | 1.269 | 1.930 | 0.831 |
| Phe Gln Arg Lys | 578.3387 | 2.7278 | 0.003 | 0.050 | 1.342 | 12.661 | 0.831 |
| 3-Methyl-L-histidine | 170.0929 | 5.9459 | 0.003 | 0.048 | 1.755 | 1.566 | 0.826 |
| Cys Arg Ser | 365.1576 | 0.7245 | 0.003 | 0.048 | 1.268 | 17.682 | 0.826 |
| Dimethylethanolamine | 90.0914 | 2.3103 | 0.003 | 0.048 | 1.217 | 4.426 | 0.826 |
| Acetylcarnitine | 204.1236 | 3.1327 | 0.003 | 0.048 | 1.163 | 2.151 | 0.826 |
| 4-Hydroxy-2-butenoic acid gamma-lactone | 85.0286 | 3.1366 | 0.004 | 0.055 | 1.174 | 2.079 | 0.815 |
| Butyryl-L-carnitine | 232.1549 | 2.1655 | 0.004 | 0.057 | 1.498 | 3.003 | 0.810 |
| Phe Gly | 223.1065 | 0.9418 | 0.005 | 0.057 | 1.396 | 2.447 | 0.805 |
| Glutarylcarnitine | 276.1446 | 4.3287 | 0.005 | 0.057 | 1.329 | 2.491 | 0.805 |
| Pro Ile | 229.1551 | 3.9085 | 0.005 | 0.057 | 1.296 | 1.749 | 0.805 |
| Pro Leu | 229.1551 | 4.3478 | 0.005 | 0.057 | 1.160 | 2.410 | 0.805 |
| Thr Thr Trp | 407.1890 | 4.0709 | 0.007 | 0.068 | 1.382 | 27.852 | 0.805 |
| Carbamoyl phosphate | 185.9807 | 0.5021 | 0.007 | 0.068 | 1.424 | 1.389 | 0.795 |
| L-Lactic acid | 89.0244 | 1.7362 | 0.007 | 0.068 | 1.137 | 1.557 | 0.795 |
| Pro Pro Gln | 363.1632 | 2.7077 | 0.009 | 0.076 | 1.380 | 19.475 | 0.795 |
| Lys Pro Asn | 358.2076 | 2.7316 | 0.009 | 0.076 | 1.374 | 16.927 | 0.792 |
| 2-(4-Methyl-5-thiazolyl)ethyl isobutyrate | 214.0897 | 0.5215 | 0.009 | 0.076 | 1.069 | 32.918 | 0.792 |
| Lys Leu Gln | 388.2547 | 0.7245 | 0.008 | 0.076 | 1.299 | 11.131 | 0.790 |
| Thr Ile Lys Glu | 490.2864 | 2.7077 | 0.011 | 0.083 | 1.357 | 11.051 | 0.785 |
| Ala Lys Leu Asp | 446.2602 | 2.7077 | 0.011 | 0.083 | 1.366 | 15.596 | 0.785 |
| Lys Ala | 218.1499 | 6.1412 | 0.011 | 0.083 | 1.314 | 2.400 | 0.779 |
| Ala Thr Ile Lys | 432.2808 | 0.7245 | 0.015 | 0.094 | 1.315 | 9.016 | 0.769 |
| D-Ribose | 149.0447 | 0.5250 | 0.015 | 0.094 | 1.262 | 1.950 | 0.769 |
| L-Alanine | 90.0555 | 4.8033 | 0.015 | 0.094 | 1.175 | 1.183 | 0.769 |
| (R)-3-Hydroxybutyric acid | 103.0398 | 1.2430 | 0.015 | 0.094 | 1.061 | 2.629 | 0.769 |
| L-Hexanoylcarnitine | 260.1858 | 1.6871 | 0.017 | 0.105 | 1.154 | 2.498 | 0.764 |
| Glu Glu | 277.1052 | 0.7207 | 0.019 | 0.112 | 1.173 | 31.304 | 0.764 |
| Val Ala Phe Asp | 451.2152 | 4.2158 | 0.024 | 0.139 | 1.363 | 13.067 | 0.754 |
| Succinic acid | 117.0188 | 1.5457 | 0.025 | 0.143 | 1.437 | 0.602 | 0.749 |
| Lys Pro Leu | 357.2499 | 5.9535 | 0.029 | 0.159 | 1.371 | 1.500 | 0.744 |
| Citrate | 191.0190 | 1.2131 | 0.033 | 0.160 | 1.460 | 1.355 | 0.738 |
| 3-Furoic acid | 111.0083 | 1.2131 | 0.033 | 0.160 | 1.363 | 1.246 | 0.738 |
| Varanic acid | 459.3071 | 3.2852 | 0.033 | 0.160 | 1.307 | 0.936 | 0.738 |
| L-alpha-Aspartyl-L-hydroxyproline | 291.0835 | 2.4590 | 0.033 | 0.160 | 1.256 | 1.530 | 0.738 |
| (+-)-Propionylcarnitine | 218.1391 | 2.5667 | 0.033 | 0.160 | 1.183 | 2.333 | 0.738 |
| 2-Methylbutyroylcarnitine | 246.1703 | 3.7269 | 0.037 | 0.173 | 1.515 | 2.204 | 0.733 |
| Tiglylcarnitine | 244.1546 | 2.0892 | 0.037 | 0.173 | 1.257 | 1.744 | 0.733 |
| Lactaldehyde | 119.0344 | 0.5450 | 0.041 | 0.186 | 1.219 | 1.738 | 0.728 |
| Taurine | 124.0070 | 4.5119 | 0.041 | 0.188 | 1.046 | 0.862 | 0.728 |
| Arg Thr Ala Arg | 525.2883 | 4.3797 | 0.046 | 0.197 | 1.375 | 19.655 | 0.723 |
| isoCitrate | 191.0190 | 0.5679 | 0.046 | 0.197 | 1.192 | 1.787 | 0.723 |

Abbreviations: SE, status epilepticus; FDR, false discovery rate; AUC, area under curve; FC, fold change; VIP, variable importance projection.
